# Supplementary material for: Targeting Tyro3 ameliorates a model of PGRN-mutant FTLD-TDP via tau-mediated synaptic pathology
Source: Nat Commun. 2018 Jan 30;9:433. doi: 10.1038/s41467-018-02821-z (PMC5789822; doi:10.1038/s41467-018-02821-z)

Supplementary information

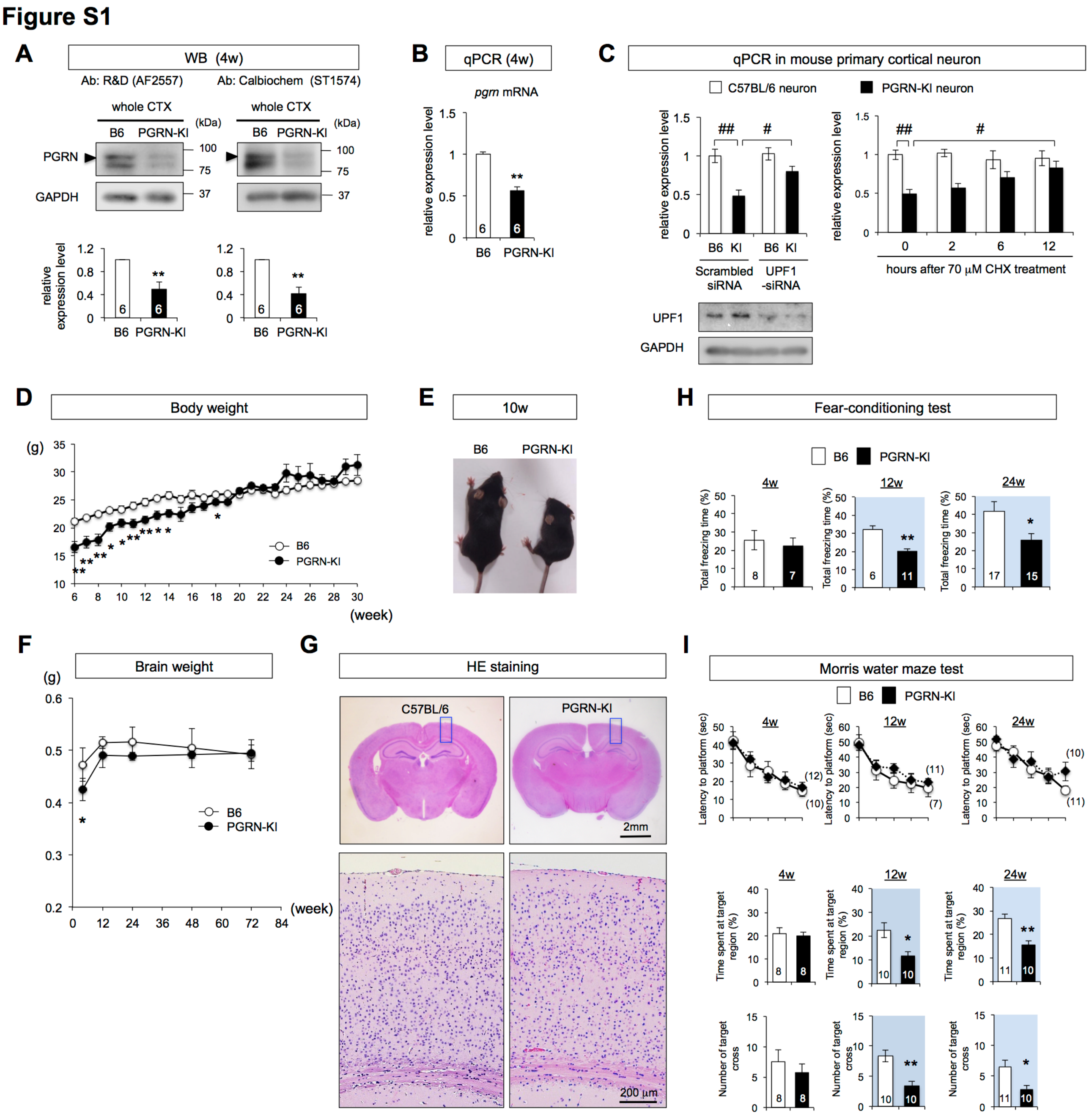

Supplementary Figure 1
PGRN expression and phenotype in PGRN-KI mice

(A) Western blot analysis revealed that PGRN protein was expressed at lower levels in PGRN-R504X-KI mice than in background mice (B6). Lower graphs show relative intensities of the PGRN band of KI and B6 mice (N=6), using two different anti-PGRN antibodies. *, $p < 0.05$, **, $p < 0.01$ (Student's t-test). Averages and S.E.M. are shown.
(B) Quantitative PCR revealed that the *Pgrn* mRNA level in cerebral cortex tissues was lower in PGRN-KI than in B6 mice (N=6). **, $p < 0.01$ (Student's t-test). Averages and S.E.M. are shown.
(C) *Upf1* siRNA and cycloheximide, which suppress nonsense-mediated RNA decay, restored the level of *Pgrn* mRNA in primary cortical neurons (E15) from KI embryos. The western blot in the lower panel confirms suppression of Upf1 by siRNA 2 days after transfection. #, $p < 0.05$, ##, $p < 0.01$ (N=4, Tukey's HSD test). Averages and S.E.M. are shown.
(D) Chronological changes in body weight in PGRN-KI and B6 mice (N=6). *, $p < 0.05$, **, $p < 0.01$ (Student's t-test). Averages and S.E.M. are shown.
(E) Images of representative mutant PGRN-KI and B6 mice.
(F) Chronological changes of brain weight in PGRN-KI and B6 mice (N=5). *, $p < 0.05$ (Student's t-test). Averages and S.E.M. are shown.
(G) Representative images of coronal sections of PGRN-KI and B6 cerebrums. Cerebral cortex is magnified. No macroscopic structural changes were observed.
(H) Fear-conditioning test revealed a difference in % total freezing time between PGRN-KI and B6 mice. Numbers of mice analyzed are shown in graph bars. *, $p < 0.05$, **, $p < 0.01$ (Student's t-test). Averages and S.E.M. are shown.
(I) In the Morris water maze test, % time spent at the target region decreased in KI mice. Latency to the platform did not differ (data not shown). Numbers of mice analyzed are shown in graph bars. *, $p < 0.05$, **, $p < 0.01$ (Student's t-test). Averages and S.E.M. are shown.

Figure S2

A

Summary of identified peptides.

Week		4			12			24		
Experiment		#1	#2	#3	#1	#2	#3	#1	#2	#3
Confidence	Total/Phospho-									
>95%	Total	92,842	52,761	65,352	74,076	42,375	49,328	73,490	44,752	31,017
	Phospho-	25,112	18,719	19,245	19,140	14,985	18,776	13,060	15,975	16,830
>90%	Total	99,051	56,328	68,691	77,171	45,417	53,627	77,609	48,073	33,532
	Phospho-	25,112	18,719	19,245	19,140	14,985	18,776	13,060	15,975	16,830
>66%	Total	111,720	65,424	75,669	84,268	52,660	63,586	87,643	56,088	39,797
	Phospho-	29,697	22,936	21,913	21,249	18,456	23,735	16,091	19,848	21,663

Summary of identified proteins.

	Week	4			12			24		
	Experiment Confidence	#1	#2	#3	#1	#2	#3	#1	#2	#3
Identified proteins	>95%	1,927	1,462	1,450	1,513	1,292	1,404	1,579	1,307	1,008
	>90%	1,999	1,497	1,492	1,550	1,324	1,435	1,641	1,341	1,047
	>66%	2,164	1,613	1,601	1,645	1,416	1,553	1,774	1,434	1,130
Phospho-proteins	>95%	1,114	896	817	808	754	796	640	765	699
iTRAQ labeled protein		1,819	1,332	1,162	1,413	1,216	1,135	1,479	1,230	901
iTRAQ labeled phospho-protein		1,058	856	653	754	724	632	605	712	624

B

p-Peptide-based PPI (q<0.05)
PGRN-KI

4w

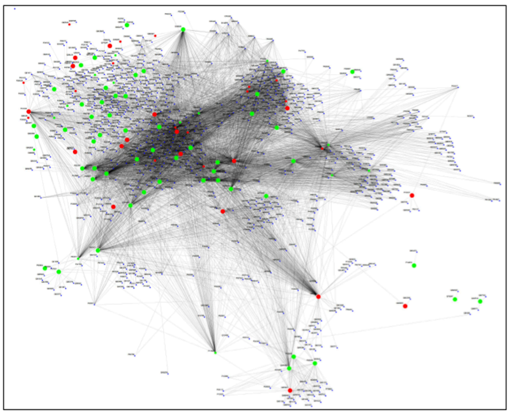

12w

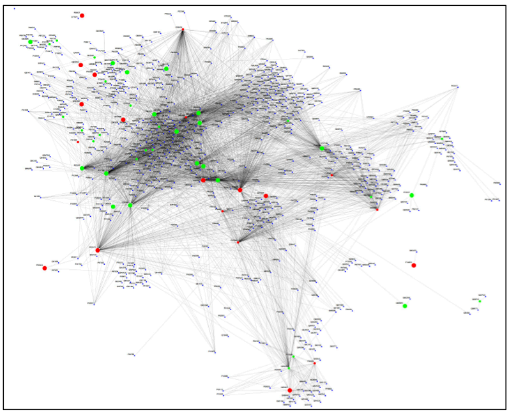

24w

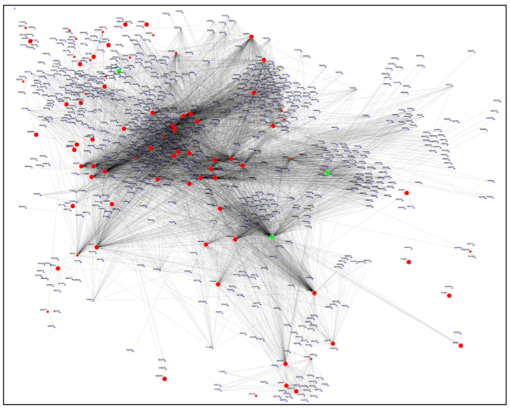

C

Signaling pathways significantly changed in PGRN-KI mice

Number of proteins corresponding to changed phosphopeptides in PGRN-KI mice were examined by Fisher's exact test for each KEGG pathway (yellow: $p<0.05$)

		KEGG Pathway	Proteins in Pathway	Number of phospho-peptides changed in PGRN-KI mice		
				4w	12w	24w
Changed	TNF-related pathways	MAPK Signaling Pathway	161	22	19	19
		mTOR Signaling Pathway	70	12	7	6
		Antigen Processing and Presentation	98	8	1	5
		Insulin related pathway	125	43	17	36
	Kinase pathway Kinase pathways	Braf-Tau related pathway	6	9	9	13
		PKC-Tau related pathway	86	34	17	32
		CaMK II-Tau related pathway	227	27	15	25
		PKA-Tau related pathway	296	32	18	31
Not changed	Kinase pathways	Rhok-Tau related pathway	281	16	10	16
		Cdk5-Tau related pathway	92	8	1	5
	TNF-related pathways	ADIPOCYTOKINE Signaling Pathway	48	0	0	0
		Alzheimer's disease	16	0	0	0
		Amyotrophic Lateral Sclerosis	17	0	0	0
		Apoptosis	54	0	0	0
		Cytokine-cytokine Receptor Interaction	6	0	0	0
		Chagas Disease	6	0	0	0
		HTLV-I Infection	11	0	0	0
		NF-kappa B Signaling Pathway	62	0	0	0
		Non-Alcoholic Fatty Liver Disease	21	0	0	0
		Osteoclast Differentiation	100	3	4	0
		TGF-Beta Signaling Pathway	15	0	0	0
		Toxoplasmosis	12	0	0	0
		Tuberculosis	20	0	0	0
		Type II Diabetes Mellitus	34	0	0	0
	TNF Signaling Pathway		109	2	0	0

D

PHOSPHO-PEPTIDE changes between PGRN-KI and WT (n=3)
in TNF and TNF-related pathways

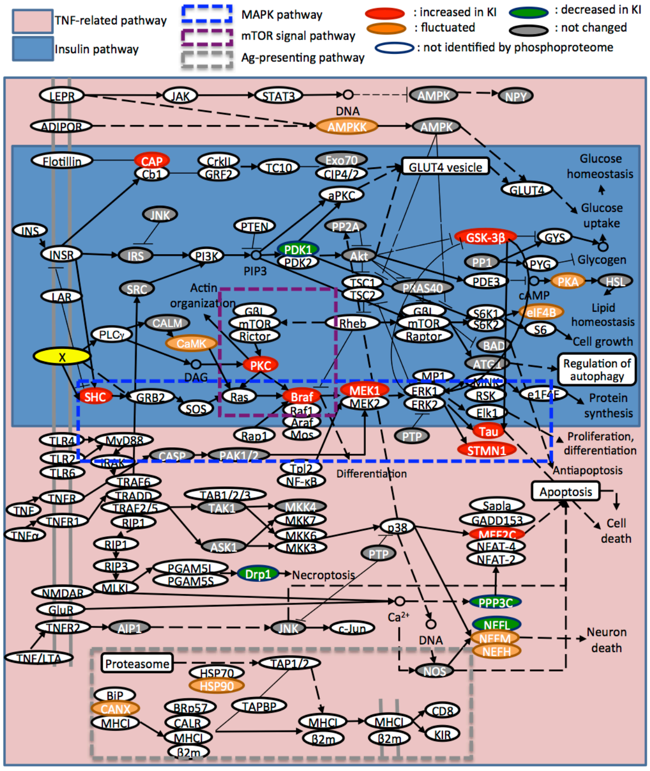

Supplementary Figure 2

Elucidation of pathological signal networks in mutant PGRN-KI mice

(A) The numbers of identified peptides or proteins from three sets of mass analyses at each age (4, 12, 24 weeks of age) are shown, along with the confidence of mass spectrometry identification (upper table). Total peptide numbers include phosphorylated and non-phosphorylated peptides (upper table). Peptide labeling with iTRAQ was used for quantitative comparison among samples. As a reference for the quality of mass spectrometry analysis, the numbers of phosphoproteins, iTRAQ-labeled proteins, and iTRAQ-labeled phosphoproteins among identified proteins deduced from phosphorylated and non-phosphorylated peptides are shown (lower table). The quantities of multiple iTRAQ tags were used to calculate the ratio of specific peptides between samples, and comparisons were performed with iTRAQ-labeled phosphopeptides that were identified at the confidence more than 95%. The change in the abundance of a phosphopeptide was judged to be statistically significant when the q-value was less than 0.05 (Welch's test with post-hoc Benjamini-Hochberg correction).

(B) Pathological PPI networks were generated by selecting nodes with the list of changed proteins that were deduced from significantly altered phosphopeptide ratios ($q<0.05$, Welch's test with post-hoc BH procedure) between C57BL/6J and mutant PGRN-KI mice (see Materials and Methods). Red and green nodes indicate proteins whose phosphorylation was increased and decreased based on the abundance of phosphopeptide(s), respectively. Node sizes reflect q-value: small ($q \geq 0.05$), medium ($0.01 \leq q < 0.05$), and large ($q < 0.01$). Edges and nodes that directly linked to the selected nodes were added secondarily (see Materials and Methods), and small nodes basically correspond to the added nodes. In the uploaded database (http://suppl.atgc.info/011/), peptide ratios and q-values are retrievable (ID: npat011, Password: hX1EKTk8).

(C) Pathological signal pathways in which the number of changed proteins (proteins with significantly altered phosphopeptide ratios) was unequally increased in comparison to that in whole proteins. KEGG pathways are marked yellow at each time point at which they were altered (Fisher's exact test, $p < 0.05$).

(D) Changed proteins were mapped to the KEGG database. Upregulated and downregulated phosphoproteins are shown in red and green, respectively. Proteins that fluctuated are shown in orange. A hypothetical candidate receptor for PGRN, which could affect PKC and MAPK signaling, is presented as "X" and mapped to an appropriate position.

Figure S3

A

Phosphopeptide changes in pathways leading tau
(q<0.05, student's t-test in PGRN-KI vs B6)

SHC-transforming protein 3
(Shc3, Q61120)

		N	Mean Ratio	p-value	q-value
S354	4w	6	1.2344	9.E-03	0.0452

Serine/threonine-protein kinase b-raf
(b-raf, P28028)

		N	Mean Ratio	p-value	q-value
S348	4w	22	1.1487	0.0065	0.0346
S348	12w	18	1.1795	0.0180	0.1108
S348	24w	10	1.3664	0.0253	0.0799
S766	12w	15	1.7508	0.0464	0.2073
S766	24w	14	3.0476	0.0041	0.0210
S769	24w	9	1.9752	0.0239	0.0779

Protein kinase C alpha type
(Prkca, P20444)

		N	Mean Ratio	p-value	q-value
S319	4w	33	1.1793	4.E-03	0.0216
S319	12w	6	1.2167	0.0327	0.0969
T497	4w	32	0.4993	3.E-07	8.E-06
T501	4w	7	0.3520	0.0002	0.0022
T638	4w	15	1.0958	0.0394	0.1268

Protein kinase C beta type
(Prkcb, P68404)

		N	Mean Ratio	p-value	q-value
S11	12w	5	0.8717	0.0041	0.0411
T500	4w	32	0.4993	3.E-07	8.E-06
T504	4w	7	0.3520	0.0002	0.0022

Protein kinase C gamma type
(Prkcg, P63318)

		N	Mean Ratio	p-value	q-value
T514	4w	32	0.4993	3.E-07	8.E-06
T518	4w	7	0.3520	0.0002	0.0022
T655	4w	137	1.2052	2.E-10	1.E-08
T655	12w	240	1.1308	2.E-09	2.E-07
T655	24w	188	1.5702	6.E-20	2.E-17
T674	12w	13	0.7673	0.0040	0.0407
S687	12w	53	0.8125	4.E-24	3.E-21
S690	4w	10	2.5918	0.0256	0.0925
S690	12w	53	0.8306	3.E-11	3.E-09

MEK1
(Map2k1, P31938)

		N	Mean Ratio	p-value	q-value
T386	4w	24	1.8079	3.E-05	0.0004

Tau
(Tau, P10637)

		N	Mean Ratio	p-value	q-value	Human
T58	4w	3	1.2883	0.0405	0.1292	T69
T170	12w	46	0.8296	0.0010	0.0148	T181
T170	24w	68	1.3169	3.E-08	1.E-06	T181
S187	12w	13	0.8524	0.0003	0.0048	S198
S188	12w	80	0.7545	7.E-10	6.E-08	S199
S188	24w	54	1.2934	8.E-06	0.0001	S199
S191	12w	103	0.9118	0.0099	0.0759	S202
S191	24w	68	1.4543	4.E-05	0.0005	S202
T194	4w	5	1.5547	0.0208	0.0802	T205
T194	12w	6	0.8294	0.0261	0.1419	T205
S203	4w	55	1.4633	2.E-09	2.E-09	S214
S203	24w	39	1.8592	6.E-10	4.E-08	S214
T220	12w	17	1.2448	2.E-05	0.0004	T231
T220	24w	19	1.2205	0.0479	0.1260	T231
S385	4w	40	0.7328	0.0037	0.0037	S396
S389	24w	27	1.2527	0.0024	0.0139	S400
T392	4w	4	2.1738	0.0028	0.0028	T403
S393	4w	146	1.2594	6.E-06	0.0001	S404
S393	24w	166	1.8318	4.E-21	1.E-18	S404
S401	24w	6	0.6361	0.0242	0.0786	S412
S402	24w	15	1.9662	0.0129	0.0492	S413
S405	4w	140	0.8756	0.0036	0.0215	S416
S405	12w	49	0.9057	0.0436	0.2018	S416
S405	24w	56	1.6927	3.E-06	6.E-05	S416

Stathmin
(Stmn1, P54227)

		N	Mean Ratio	p-value	q-value
S25	4w	195	1.0972	5.E-05	0.0006
S25	12w	220	1.0551	0.0035	0.0374
S25	24w	61	1.9559	2.E-13	2.E-11
S38	24w	362	1.3896	2.E-14	3.E-12
S46	24w	103	1.1061	0.0183	0.0638

Glycogen synthase kinase-3 beta
(Gsk-3β, Q9WV60)

		N	Mean Ratio	p-value	q-value
S215	4w	82	1.3060	2.E-08	6.E-07
S215	24w	32	1.4019	2.E-07	6.E-06
S216	4w	19	1.4844	0.0010	0.0079
S219	12w	6	1.4789	0.0032	0.0198
S389	12w	9	1.2978	0.0213	0.0813

Myocyte-specific enhancer factor 2C (Mef2c, Q8CFN5)

		N	Mean Ratio	p-value	q-value
S222	24w	8	1.3945	0.0036	0.0189

Sorbin and SH3 domain-containing protein 1
(CAP / Sorbs1, Q62417)

		N	Mean Ratio	p-value	q-value
S345	24w	5	1.3579	0.0057	0.0269

B

Domain structures and length of Tau isoforms

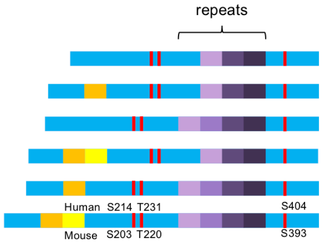

A.A. lengths of isoforms

Isoform	Human	Mouse
0N3R	352	341
1N3R	381	370
0N4R	383	372
2N3R	410	399
1N4R	412	401
2N4R	441	430

Positions of phospho-sites of interest in human Tau isoforms

isoform	A.A. length	human Tau phospho-sites corresponding to mouse Tau S203 and T220		RefSec Accession No.		Uniprot ID
		S203 (mouse)	T220 (mouse)	protein	mRNA	
2N/3R	410	S214	T231	NP_001190181.1.	NM_001203252.1.	P10636-5
2N/4R	441	S214	T231	NP_005901.2.	NM_005910.5.	P10636-8
1N/3R	381	S185	T202	NP_001190180.1	NM_001203251.1	P10636-4
1N/4R	412	S185	T202	NP_001116539.1.	NM_001123067.3.	P10636-7
0N/3R	352	S156	T173	NP_058525.1.	NM_016841.4.	P10636-2
0N/4R	383	S156	T173	NP_058518.1.	NM_016834.4.	P10636-6

Positions of phospho-sites of interest in isoforms of mouse Tau

isoform	A.A. length	Corresponding positions of the phospho-sites in mouse Tau isoforms		RefSec Accession No.		Uniprot ID
		S203	T220	protein	mRNA	
2N/3R	399	S203	T220	XP_006532471.1	XM_006532408.3	-
2N/4R	430	S203	T220	NP_001033698.1.	NM_001038609.2.	P10637-2
1N/3R	370	S174	T191	XP_006532472.1	XM_006532409.2	-
1N/4R	401	S174	T191	XP_006532470.1	XM_006532407.3	-
0N/3R	341	S145	T162	NP_001272385.1.	NM_001285456.1.	P10637-4
0N/4R	372	S145	T162	NP_034968.3.	NM_010838.4.	P10637-5

D

TAR DNA-binding protein 43
(TDP43, Q921F2)

		N	Mean Ratio	p-value	q-value
S2	4w	2	0.8807	0.8409	---
T141	4w	1	1.0412	---	---
S144	4w	1	4.2295	---	---
S163	4w	1	1.7172	---	---
S258	4w	2	2.0399	0.7199	---
S273	4w	1	0.2965	---	---
S292	4w	1	0.2537	---	---

Tyrosine-protein kinase receptor TYRO3
(Tyro3, P55144)

		N	Mean Ratio	p-value	q-value
S100	4w	1	1.4733	---	---
T115	4w	1	2.1805	---	---
S847	4w	1	2.5796	---	---

Myristoylated alanine-rich C-kinase substrate
(Marcks, P26645)

		N	Mean Ratio	p-value	q-value
S27	4w	154	1.2508	0.0187	0.0798
S27	24w	144	1.4284	0.0007	0.0061
S29	4w	10	0.6195	0.0086	0.0452
S29	12w	52	1.2483	0.0000	1.E-06
S46	12w	106	1.0837	0.0366	0.1283
S122	4w	55	0.8900	0.0012	0.0097
S124	4w	15	0.8208	0.0267	0.1016
S125	4w	34	0.7919	0.0000	0.0004
S125	24w	6	1.2452	0.0367	0.1285
S128	4w	42	0.7629	0.0000	5.E-05
S138	4w	74	0.7694	0.0033	0.0223
S138	24w	38	1.1970	0.0491	0.1550
S140	4w	15	0.6864	0.0008	0.0076
T143	24w	50	1.2296	0.0124	0.0594
S163	4w	67	0.6101	0.0003	0.0029
S163	24w	8	1.4870	0.0006	0.0061
S171	4w	27	0.6383	0.0001	0.0014

C

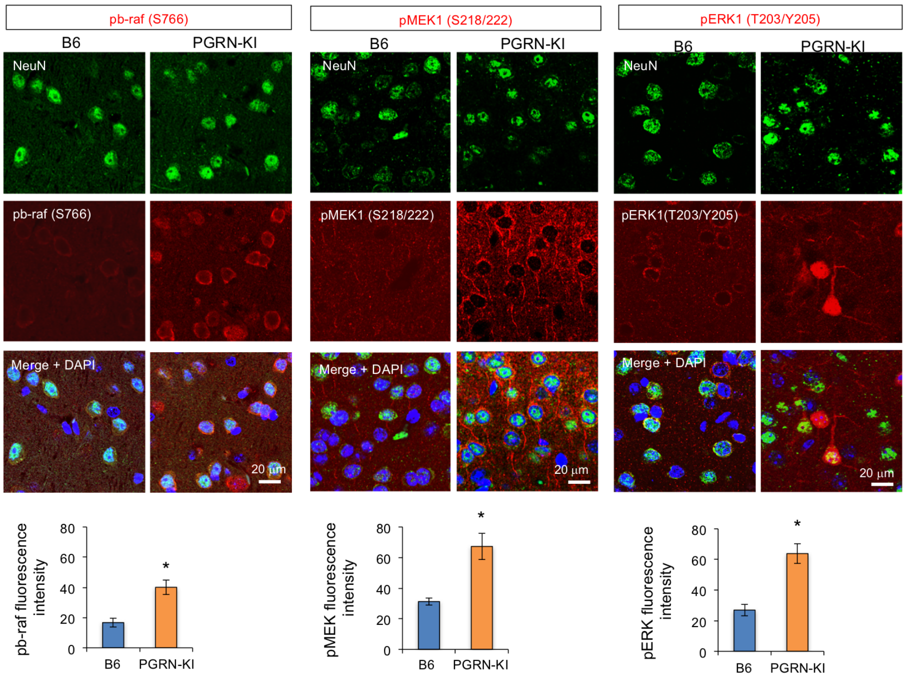

Supplementary Figure 3
Changes in protein phosphorylation in tau-related pathways detected by mass spectrometry

(A) Summary of phosphorylation changes in signal proteins in the pathway leading to tau phosphorylation. Mean peptide ratio and number of peptide peaks detected in mass analysis (N) are shown, as are p-values and q-value (Welch's test with post-hoc Benjamini-Hochberg correction) for the comparison between PGRN-KI and B6 mice. In the uploaded database (http://suppl.atgc.info/011/), all phosphopeptide data (ratios and q-values) are retrievable (ID: npat011, Password: hX1EKTk8).

(B) Upper schemes show multiple isoforms of human and mouse tau proteins. Critical phosphorylation sites addressed in this study and their correspondences between human and mouse are indicated. Lower panels show database accession numbers of human and mouse tau isoforms.

(C) Immunohistochemistry of phospho-B-Raf (Ser766), phospho-MEK1 (Ser218/222), and phospho-ERK1 (Thr203/Tyr205) revealed cytoplasmic stains in M2 cortex of PGRN-KI mice at 4 weeks of age. Lower graphs show quantitation of the stain signals. The mean values of signal intensities from 120 cells randomly selected in M2 frontal cortex of three mice were statistically evaluated by Student's t-test (N=3,*$p < 0.05$). Averages and S.E.M. are shown.

(D) Phosphorylated peptides of TDP43, Tyro3, and MARCKS identified by mass spectrometry. Because the numbers of detected peaks (N) for TDP43 and Tyro3 were small, the change could not be confirmed statistically.

Figure S4

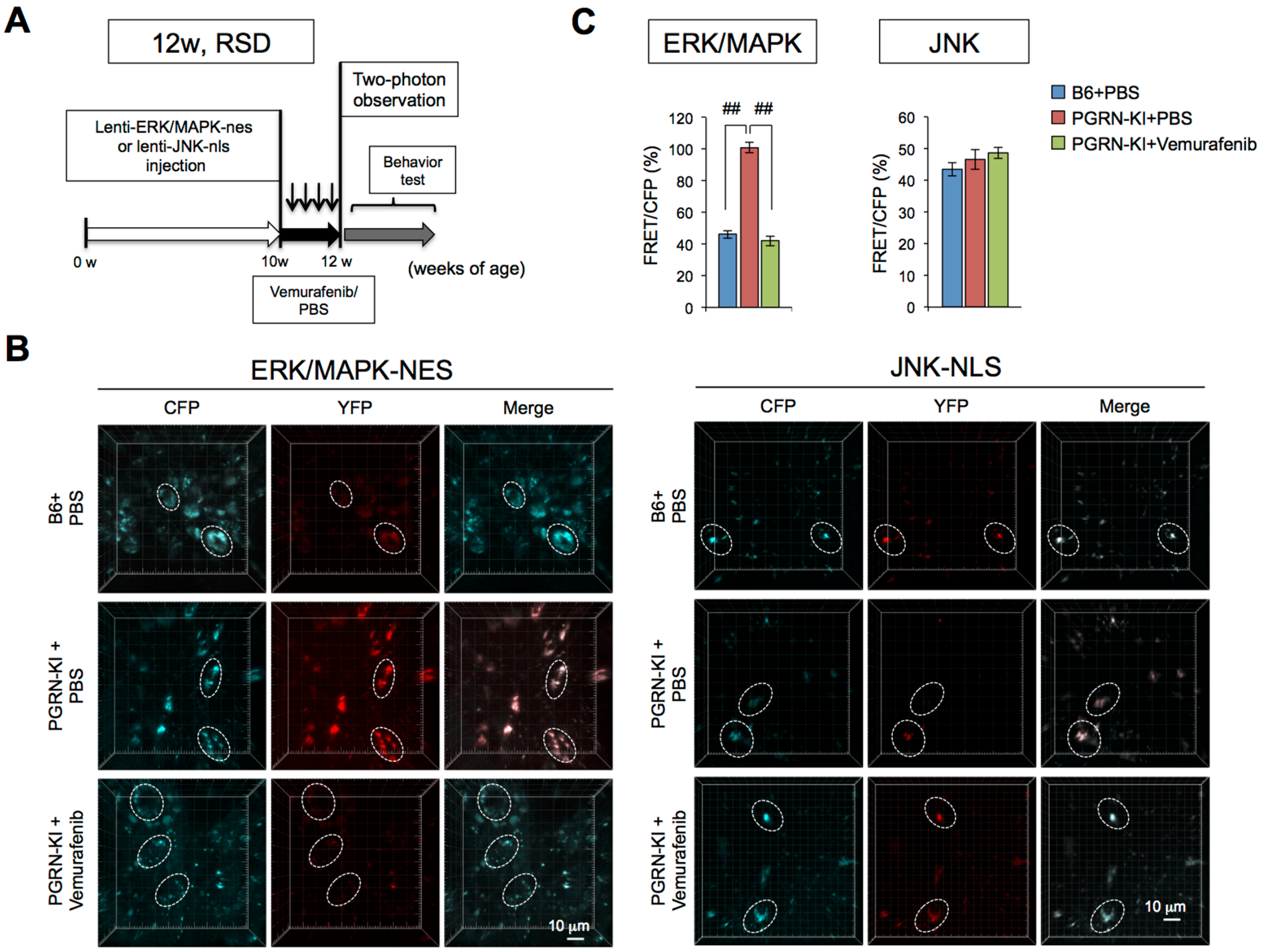

Supplementary Figure 4
***In vivo* imaging of MAPK activation in the cortex of PGRN-KI mice**

(A) Protocol for *in vivo* imaging of MAPK activation. Lentiviral vectors expressing MAPK or JNK substrate for FRET were infected into RSD 2 weeks before observation at layer 2 of M2 cortex by two-photon microscopy. Vemurafenib or PBS was injected intrathecally to mice for 1 week before observation.

(B) Actual images of FRET acquired from B6+PBS, PGRN-KI+PBS, and PGRN-KI+vemurafenib mice. Dotted circle indicates a single neuron. FRET from CFP to YFP of the ERK/MAPK substrate was higher in PGRN-KI+PBS than in B6+PBS. The abnormal increase in FRET was rescued in PGRN-KI+vemurafenib. FRET of the JNK substrate was not altered in PGRN-KI+PBS.

(C) Quantitative analyses of FRET signals of ERK/MAPK and JNK substrates of B6+PBS, PGRN-KI+PBS, and PGRN-KI+vemurafenib mice. P-values were computed by Tukey's HSD test. ##, $p < 0.01$. Averages and S.E.M. are shown.

Figure S5

A

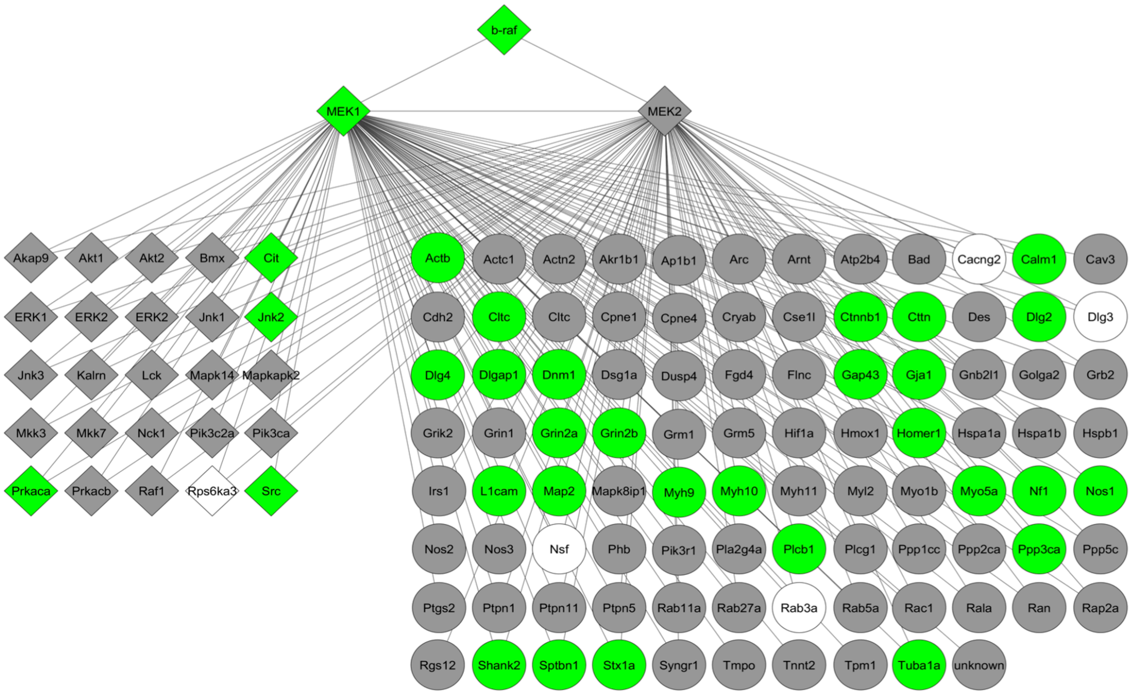

B

phosphorylated proteins	suppression by Vemurafenib	Braf independent	Braf downstream	Fisher's exact test p-value
	suppressed proteins	479	33	
	detected proteins	1533	38	p=5.69E-05 **
	ratio	0.312	0.868	

phosphorylation sites	suppression by Vemurafenib	Braf independent	Braf downstream	
	suppressed p-sites	882	133	
	detected p-sites	5904	401	p=1.64E-12 **
	ratio	0.149	0.332	

Supplementary Figure 5

(A) Phosphorylation states of upstream and downstream molecules of B-Raf in PGRN-KI mice after treatment with vemurafenib. Phosphoproteins detected with > 95% confidence in mass spectrometry of whole cerebral cortex from PGRN-KI mice were examined for changes relative to vemurafenib-treated PGRN-KI mice. Green indicates proteins whose phosphorylation was suppressed in vemurafenib-treated PGRN-KI mice with a q-value less than 0.05, and white indicates statistically unchanged proteins. Diagonal and circular nodes indicate kinase and non-kinase proteins, respectively. Gray indicates proteins not detected by mass spectrometry with > 95% confidence. The original data from the phosphoproteome analysis are available at http://suppl.atgc.info/011/ (ID: npat011, Password: hX1EKTk8).

(B) Numbers of phosphorylated proteins and phosphorylation sites suppressed by vemurafenib are shown in the B-Raf-downstream or outside of the downstream. Their ratios to the total detected numbers are calculated in the two categories, and their difference is examined by Fisher's exact test.

Figure S6

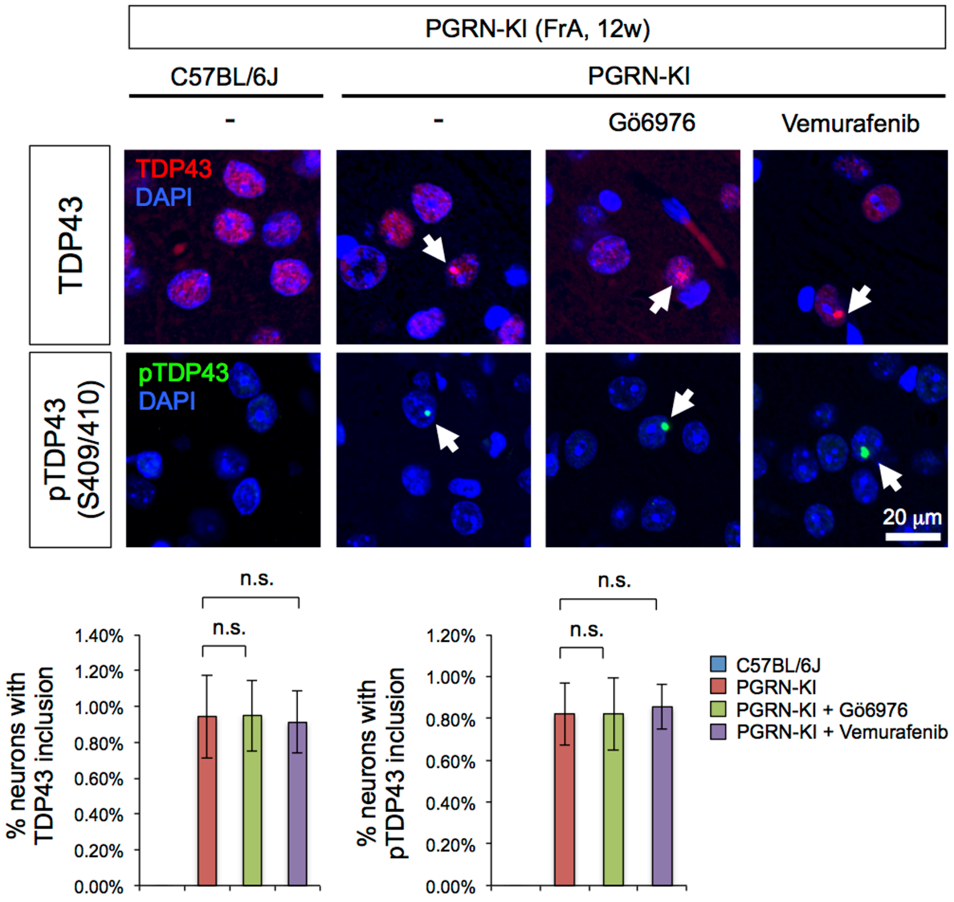

Supplementary Figure 6

Improved spine pathology and cognitive symptoms are not associated with TDP43 aggregation

Gö6976 and vemurafenib treatment, which ameliorate spine pathology and cognitive impairment, did not reduce the percentage of TDP43 aggregate–positive cells in PGRN-KI mice at 12 weeks. Upper, middle, and lower panels show representative images of TDP43 aggregates in PGRN-KI and B6 mice. In TDP43 and pTDP43 immunohistochemistry, no significant difference in the percentage of aggregate-positive cells was detected between non-treated and treated PGRN-KI mice (Tukey's HSD test). Averages and S.E.M. are shown.

Figure S7

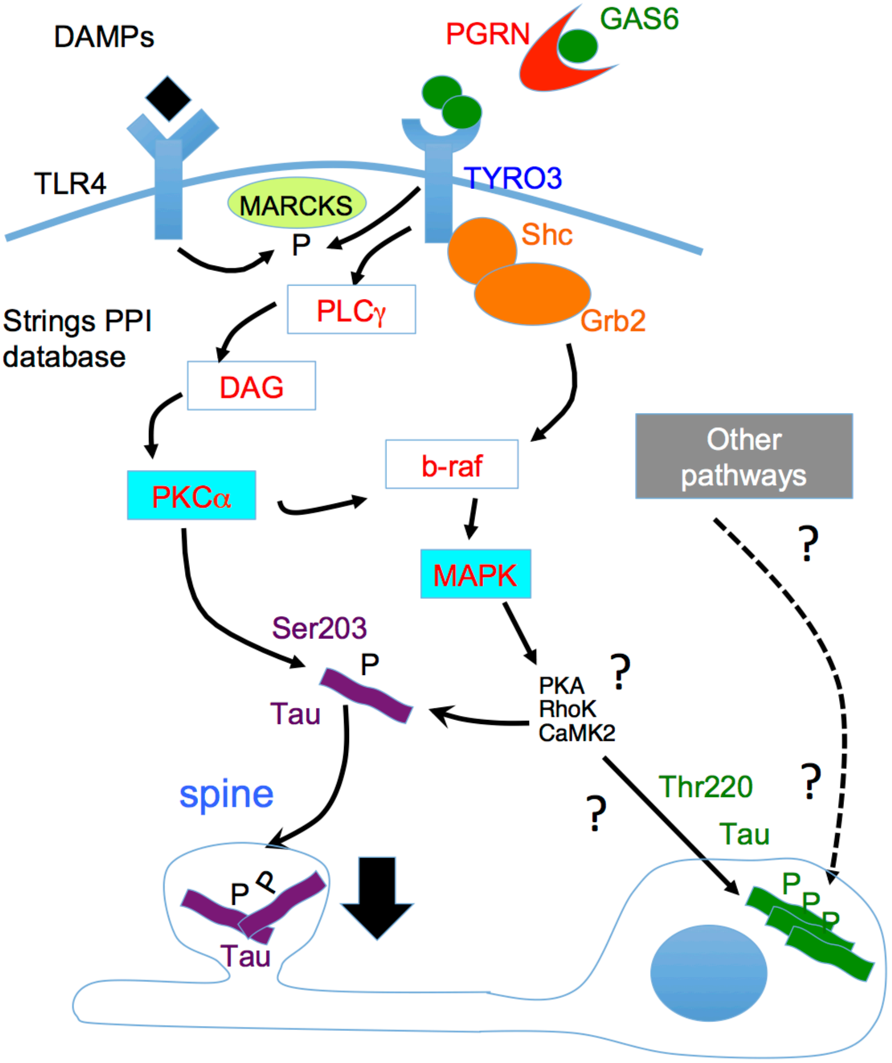

Supplementary Figure 7
Scheme of molecular pathways in PGRN-linked FTLD
The absence of PGRN-mediated suppression of Gas6–Tyro3 binding activates the PLCγ–DAG–PKC pathway and MAPK signaling. Consequently, tau proteins are phosphorylated at two positions (Ser203 and Thr220), resulting respectively in two different pathologies, TDP43 mislocalization and cytoplasmic accumulation.

Supplementary Figure 8
Full scan images of blots.

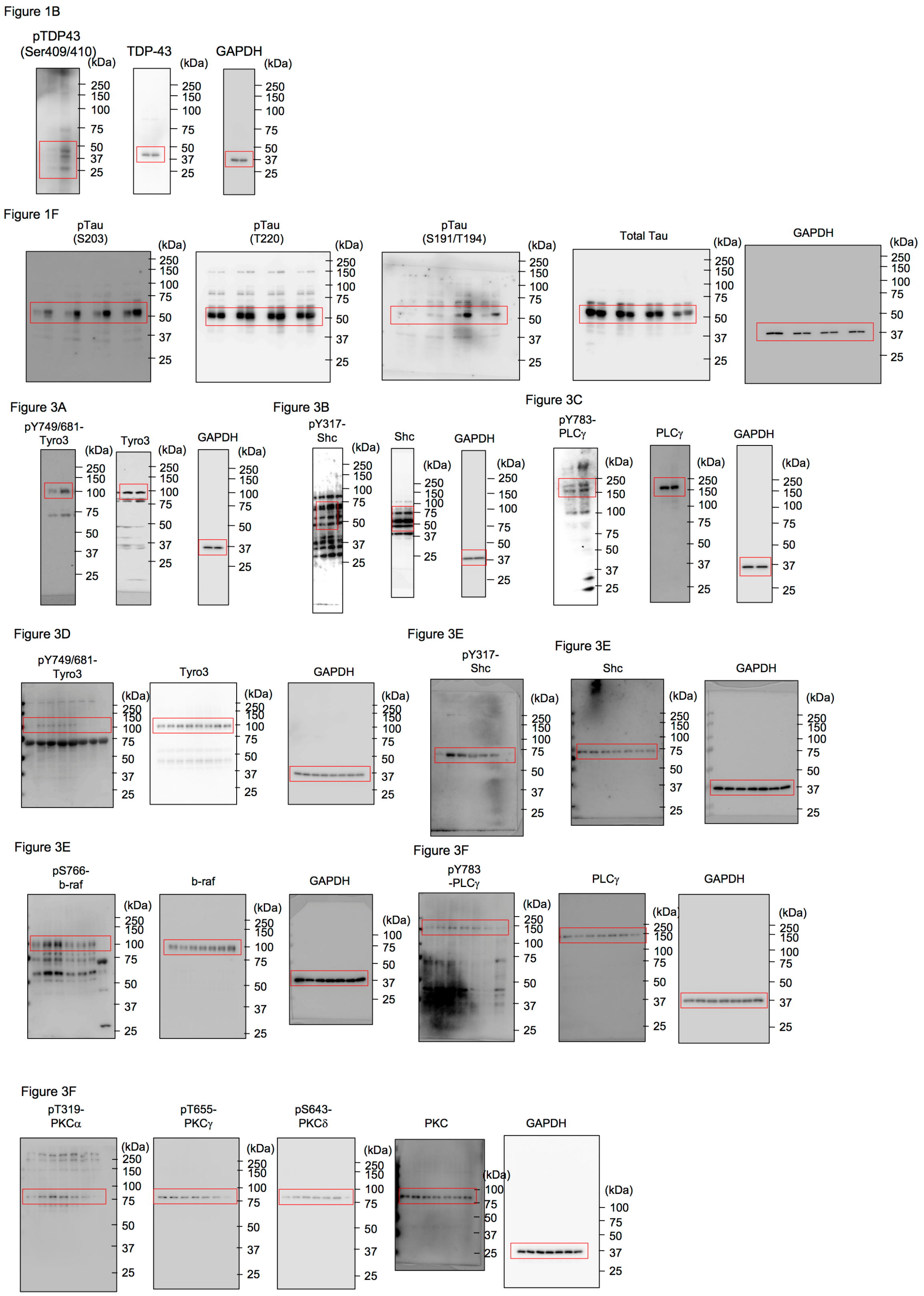

Figure 4

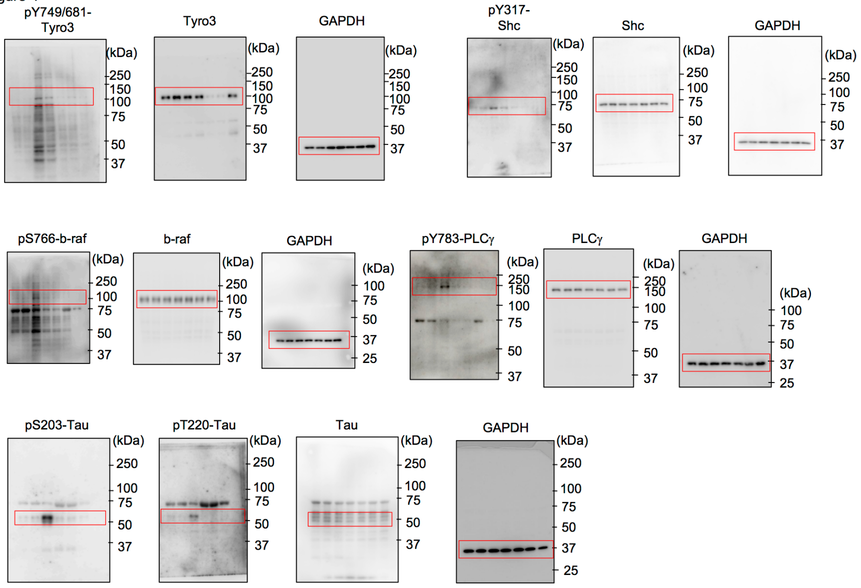

Figure 6B

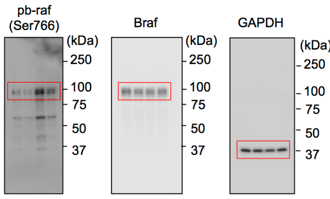

Figure 6C

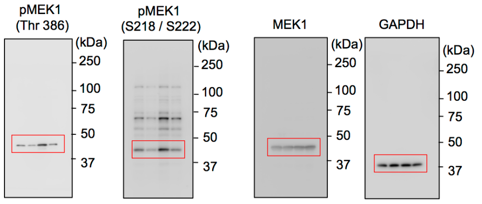

Figure 6D

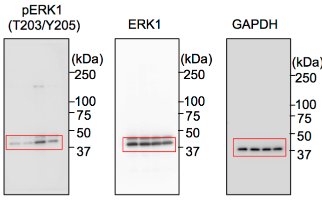

Figure 6E

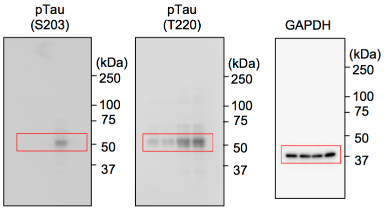

Figure 7B

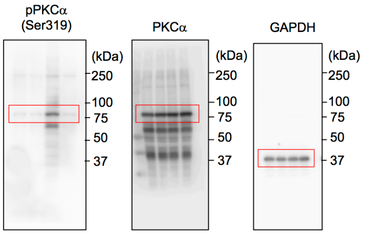

Figure 7C

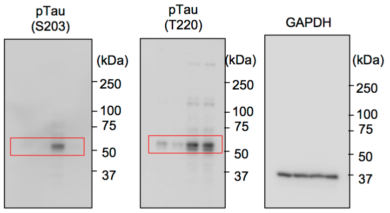

Figure 9F

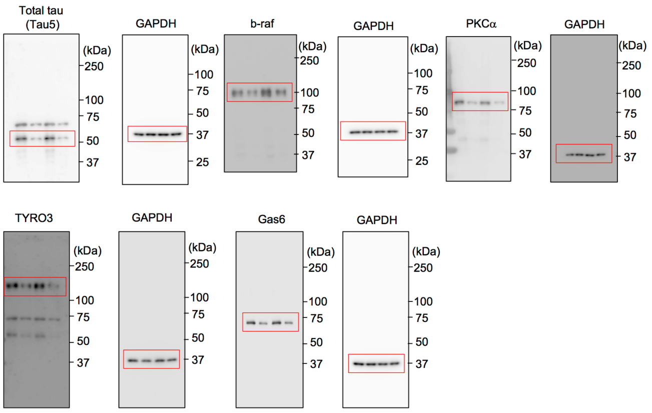

Supplement: Supplementary file 1 — Supplementary Information [file 41467_2018_2821_MOESM1_ESM.pdf]
