# Supplementary material for: Targeting Tyro3 ameliorates a model of PGRN-mutant FTLD-TDP via tau-mediated synaptic pathology
Source: Nat Commun. 2018 Jan 30;9:433. doi: 10.1038/s41467-018-02821-z (PMC5789822; doi:10.1038/s41467-018-02821-z)
Supplement: Supplementary file 3 — Description of Additional Supplementary Files [file 41467_2018_2821_MOESM3_ESM.pdf]

Description of Additional Supplementary Files

File Name: Supplementary Data 1
Description: Source data for graphs.
